# Correction: HLA Haplotyping from RNA-seq Data Using Hierarchical Read Weighting

**DOI:** 10.1371/journal.pone.0104664

**Published:** 2014-07-31

**Authors:** The PLOS ONE Staff

The term haplotype is used incorrectly throughout the manuscript. The correct term should be type.

The term haplotype is used incorrectly in the article title. The correct title is: HLA Typing from RNA-seq Data Using Hierarchical Read Weighting. The correct citation is: Kim HJ, Pourmand N (2013) HLA Typing from RNA-seq Data Using Hierarchical Read Weighting. PLoS ONE 8(6): e67885. doi:10.1371/journal.pone.0067885
